# Application of Compositional Data Analysis to Study the Relationship between Bacterial Diversity in Human Faeces and Sex, Age, and Weight

**DOI:** 10.3390/biomedicines11082134

**Published:** 2023-07-28

**Authors:** Elio López-García, Antonio Benítez-Cabello, Antonio Pablo Arenas-de Larriva, Francisco Miguel Gutierrez-Mariscal, Pablo Pérez-Martínez, Elena María Yubero-Serrano, Francisco Noé Arroyo-López, Antonio Garrido-Fernández

**Affiliations:** 1 Food Biotechnology Department, Instituto de la Grasa (CSIC), Carretera Utrera km 1, Campus Universitario Pablo de Olavide, Building 46, 41013 Seville, Spain; elopez@ig.csic.es (E.L.-G.); fnoe@ig.csic.es (F.N.A.-L.); agarrido@ig.csic.es (A.G.-F.); 2 Unidad de Gestión Clínica Medicina Interna, Lipids and Atherosclerosis Unit, Maimonides Institute for Biomedical Research in Córdoba, Reina Sofia University Hospital, University of Córdoba, 14004 Córdoba, Spain; aparenaslarriva@gmail.com (A.P.A.-d.L.); francisco.gutierrez@imibic.org (F.M.G.-M.); pablopermar@yahoo.es (P.P.-M.); elena.yubero@imibic.org (E.M.Y.-S.); 3 CIBER Physiopathology of Obesity and Nutrition (CIBEROBN), Institute of Health Carlos III, 28029 Madrid, Spain

**Keywords:** clinical trial, metataxonomic analysis, bacterial population, human stool

## Abstract

This work uses Compositional Data Analysis (CoDA) to examine the typical human faecal bacterial diversity in 39 healthy volunteers from the Andalusian region (Spain). Stool samples were subjected to high-throughput sequencing of the V3 and V4 regions of the 16S ribosomal RNA gene using Illumina MiSeq. The numbers of sequences per sample and their genus-level assignment were carried out using the Phyloseq R package. The alpha diversity indices of the faecal bacterial population were not influenced by the volunteer’s sex (male or female), age (19–46 years), and weight (48.6–99.0 kg). To study the relationship between these variables and the faecal bacterial population, the ALDEx2 and coda4microbiome CoDA packages were used. Applying ALDEx2, a trend suggesting a connection between sex and the genera *Senegalimassilia* and *Negatibacillus* (slightly more abundant in females) and *Desulfovibrio* (more abundant in males) was found. Moreover, age was tentatively associated with *Streptococcus*, *Tizzerella*, and *Ruminococaceae*_UCG-003, while weight was linked to *Senegalimassilia.* The exploratory tool of the coda4microbiome package revealed numerous bacterial log-ratios strongly related to sex and, to a lesser extent, age and weight. Moreover, the cross-sectional analysis identified bacterial signature balances able to assign sex to samples regardless of controlling for volunteers’ age or weight. *Desulfovibrio*, *Faecalitalea*, and *Romboutsia* were relevant in the numerator, while *Coprococcus*, *Streptococcus*, and *Negatibacillus* were prominent in the denominator; the greater presence of these could characterise the female sex. Predictions for age included *Caproiciproducens*, *Coprobacter*, and *Ruminoclostridium* in the numerator and *Odoribacter*, *Ezakiella*, and *Tyzzerella* in the denominator. The predictions depend on the relationship between both groups, but the abundance of the first group and scarcity of the second could be related to older individuals. However, the association of the faecal bacterial population with weight did not yield a satisfactory model, indicating scarce influence. These results demonstrate the usefulness of the CoDA methodology for studying metagenomics data and, specifically, human microbiota.

## 1. Introduction

The human gastrointestinal tract takes up 250–400 m^2^, providing habitat for many microorganisms (>10^14^ cells), collectively weighing approximately 2–3 kg. This microbiota offers numerous benefits, such as harvesting energy, strengthening gut integrity, regulating the immunity system, and protecting against pathogens, among other physiological functions. However, the mechanisms of these activities could be disrupted due to an altered gut microbial composition, a process known as dysbiosis [1]. Therefore, using appropriate methodologies to study the human gut microbiome is critical.

Analysis of microbiome data sets has typically been performed using the standard multivariate statistics developed for the Euclidean space. However, researchers have advised that specific metadata characteristics could resemble compositional data (CoDa) [2]. For instance, the total number of recorded counts is irrelevant; absolute and relative frequencies carry the same information about the composition. Moreover, analysing subsets of the original microbial taxa is expected to be compatible with the principles of scale invariance and sub-compositional coherence in CoDa analysis. Based on these premises, statistical studies of the microbiome should follow CoDA procedures and, particularly, employ the Cartesian coordinates (*ilr* coordinates) that enable the application of the classical methods [2].

Erb and Notredame [3] highlighted that the correlation and ubiquity of gene expression analyses lacked an objective criterion. The necessity for normalisation could be circumvented by employing a relative approach known as log-ratio analysis. This method enables the identification of proportional gene pairs from un-normalised data. The same authors demonstrated that using an unaltered gene as a reference (additive log-ratio transformation, *alr*) improved the sensitivity. Additionally, these authors explored the relationship between proportionality and partial correlation, deriving expressions to evaluate it. Moreover, Gloor et al. [4], in their publication “It’s all relative: analysing microbiome data as compositions”, devoted to the study of the human microbiome tongue versus mucosa data set, definitively demonstrated that the CoDA approach could be readily scalable to microbiome-sized analysis. They provided example codes and recommendations for improving the analysis and reporting the results. That paper also offers a comprehensive justification for considering such data as compositions and explains the fundamental principles for applying the new CoDA statistical tool. This perspective was further elaborated upon in a review by Gloor et al. [5], wherein investigators were alerted to the risks associated with disregarding the compositional nature of the data. They emphasised that high-throughput sequencing (HTS) microbiome data sets can and should be statistically analysed as compositions. Subsequently, Quinn et al. [6] reviewed the principles of CoDA, presented evidence regarding the compositional structure of sequencing data, discussed the available methods, and highlighted directions for the future in this field. Fortunately, implementing CoDA methodologies in microbiome analysis has received significant support and development [7,8,9]. There has also been a focus on examining differences in taxon abundance between two classes of subjects or samples to characterise different ecological niches [10].

Developments in CODA tools have been dedicated to various aspects, including ANOVA-Like Differential Expression (ALDEx) Analysis for Mixed Population RNA-Seq [11], the utilisation of linear and nonlinear correlation estimators to reveal undocumented taxa interactions in microbiome data [12], and bias correction in analysis compositional analysis of microbiomes [13]. A recent critical review of procedures for studying microbiome differential abundance methods found that different methodologies yield varying results across 38 data sets [14]. Recently, the application of CoDa analysis to study the composition of the human gut bacterial composition and the impact of probiotic intake has also been carried out by López-García et al. [15].

The objective of this study was to utilise the CoDA methodology to analyse the usual bacterial makeup of human faeces among a group of healthy volunteers. Furthermore, this study aimed to explore how this composition relates to factors such as sex, age, and weight.

## 2. Materials and Methods

### 2.1. Human Clinical Trial

Complete details of the clinical trial conducted in the present work can be found in López-García et al. [15]. This study followed the Declaration of Helsinki and was conducted at the Maimónides Biomedical Research Institute (IMIBIC, Cordoba, Spain) and the Reina Sofía University Hospital (Cordoba, Spain) between July and September 2021. The intervention protocol was approved by the Human Investigation Review Committee of the Reina Sofia University Hospital with reference 2519-N-20, following institutional and Good Clinical Practice Guidelines. Informed consent was obtained from all subjects involved in this study.

A total of 39 healthy volunteers from Seville (*n* = 6) and Córdoba (*n* = 33) provinces (Andalusian, Spain) fulfilled the inclusion and exclusion criteria and were selected to participate in this study. Participants’ inclusion and exclusion criteria, baseline clinical parameters, and the participants’ metabolic and lipid profiles can be consulted in López-García et al. [15]. Descriptive statistics analysis for sex, age, and weight variables of the 39 selected volunteers are shown in Table 1.

### 2.2. Stool Sample Processing

A total of 39 faecal samples, one for each participant, were processed at the beginning of the clinical trial. For this purpose, an aliquot of 250 mg of each faecal sample was homogenised in saline solution (0.9% NaCl) using DNA/RNA ShieldTM faecal collection tubes (Zymo Research, Irvine, CA, USA). Then, bacterial DNA from human faecal samples was purified and isolated using the ZymoBIOMICSTM DNA/RNA Miniprep kit (Zymo Research, Irvine, CA, USA) and frozen at −20 °C until further analysis. Prior to massive sequencing, DNA concentration was measured using a Qubit 4 fluorometer (Thermo Fisher Scientific, Geel, Belgium), always reaching values above 5 ng/µL.

### 2.3. Analysis of Bacterial Diversity

Metataxonomic analysis of the 39 faecal samples was carried out as described in López-García et al. [15]. Briefly, to determine bacterial populations, the V3 and V4 regions (459 bp) of the 16S ribosomal RNA gene were sequenced in an Illumina MiSeq sequencing system at FISABIO facilities (Valencia, Spain). Metataxonomics data under default parameters were primarily analysed using the R package phyloseq 1.32.0 [16]. For each sample, bacterial Amplicon Sequence Variant (ASV) were retained; the remaining reads were clustered against those ASVs, allowing one mismatch to correct for error sequencing. Bacterial taxonomy at the genus level was assigned using the SILVA 138 SSU database [17]. Then, the Past 4.13 software [18] was used to determine alpha diversity indices.

### 2.4. CoDa Analysis

The experimental setup was divided into two sections. The initial one provided details about the experimental conditions, while the subsequent section focused on the abundance of the microbiome. Thus, the matrix consisted of *n* rows, each corresponding to one of the 39 volunteers, and *k* columns representing environmental variables (sex, age, and weight) followed by the bacterial taxa (ASVs) derived from metataxonomic analysis. The data analysis considered both sections together or individually. In the section on faeces’ bacterial composition, each cell *xij* indicates the number of sequences (reads) associated with taxon *j* in sample *i*. Key characteristics of the bacterial abundance matrix were (i) the total counts (sequences) varied significantly among participants, (ii) the count was limited by the maximum number of sequences read by the sequencer, and (iii) the matrix contained a high proportion of zeros. Treating the dataset as compositional eliminated the need for the normalisation pre-processing step typically used to address the first issue [5,6,8].

Regarding the second aspect, increasing the abundance of one bacterial taxon decreased the counts of others due to the total count constraint imposed by the equipment. CoDA effectively handled this feature. Finally, the high proportion of zeros (sparsity) was treated as zero counts and replaced with imputed values using the Bayesian Multiplicative (B.M.) method proposed by Martín-Fernández et al. [19] or replaced by adding a value of 1 in the abundance matrix. Rivera-Pinto [7] compared both ways and found somewhat similar results. In this study, they were used as recommended by each package. The advantageous conditions a proper CoDA provides are permutation invariance, scale invariance, and sub-compositional coherence. These valuable characteristics are particularly interesting since one usually works with sub-compositions [5,6,8].

The sequential steps of CoDa analysis executed in this study were as follows. Firstly, the data were subjected to an assessment of alpha diversity using the parameters provided by Past software [18]. Next, the ALDEx2 program [11] was utilised to investigate the influence of sex, age, and weight on the composition of the human faeces bacteria. This program accounts for the compositional structure of microbiome data and uses the Dirichlet-multinomial model to examine differences between counts (sequences) or relationships between bacterial taxa and experimental conditions. The procedure incorporates biological and sampling variation to calculate the expected false discovery rate (FDR) given the variation, based on a Wilcoxon Rank Sum test and Welch’s *t*-test (via aldex.ttest), a Kruskal–Wallis test (via aldex.kw), a generalised linear model (via aldex.glm) or a correlation test (via aldex.corr). All tests report *p*-values, and Benjamini–Hochberg corrected *p*-values. Interestingly, ALDEx2 can also be employed to investigate complex designs, such as in this work, which includes three variables, sex (binary), age (continuous), and weight (continuous). In ALDEx2, considering the CoDa structure, the initial step involves calculating the *clr* (log-ratio of each component over the geometric mean of all components, on an observation-wise basis). Subsequently, various tests are applied to the *clr* transformed data set, yielding both the regular *p*-values and the Benjamini–Hochberg corrected *p*-values. The analysis is conducted using the aldex.glm function, which uses a probabilistic compositional approach. The returned results provide the expected values of the glm function for the input variables included in the model. The glm.test output presents the estimated coefficients for each variable, their standard error, *p*-value and the Holm–Bomberroni adjusted *p*-values for the effect of each variable.

Concerning the usage of coda4microbiome [9], the package was employed to predict sex, age or weight as a function of log-ratios of taxa. The package comprises several functions for exploring and studying microbiome data. While it was primarily developed for identifying biomarkers for disease diagnosis, its exploratory, cross-sectional, and longitudinal modules offer multiple potential applications. The exploratory analysis was conducted using the explore.logratios function, which explores the association of each pairwise log-ratio with a dependent variable (binary or continuous; in this case, sex, age, and weight), with or without co-variables. The importance of this analysis is based on the principle that when a taxon is highly associated with the outcome in the environment, any log-ratio of it is likely to be associated with such outcome, regardless of the second taxon. The association of each taxon with the dependent variable is assessed using the prediction accuracy (logistic regression for binary variables) or the Pearson correlation coefficient (continuous variables). When the tool is applied to one or both of the other two conditions as co-variables, it also provides an indirect evaluation of their possible influences on the predictions of the primary variable. The cross-sectional study complements the exploratory findings. It was performed for each variable directly or after removing the effect of one or the other two variables by using them as co-variables. These studies aim to demonstrate not only the possibility of correctly assigning sex, age, or weight based on taxonomic balances even in the absence of significant individual differences between them but indirectly suggest that these body conditions can modify the gut microbiome in humans.

ALDEx2 and coda4microbiome R packages were run in R v 4.3.0 [20], under “RStudio 2023.03.1 + 446|Release 2023-05-12” for Windows [21].

## 3. Results

This study aimed to identify the typical bacterial composition of human faeces found in 39 healthy individuals and examine their relationship with sex, age, and weight. For this purpose, faecal samples collected from a previous study conducted in Seville and Cordoba (Andalusia, Spain) were utilised. The original study was designed to assess the probiotic potential of *Lactiplantibacillus pentosus* LPG1 [15]. All samples analysed in this survey were obtained before the probiotic administration (baseline conditions) and subjected to a culture-independent analysis of bacterial DNA, which showed the presence of both viable and non-viable microorganisms in faeces. The participant group consisted of 39 (*n*) volunteers, comprising 20 females and 19 males, with an average age of 32.51 ± 7.64 years (ranging from 19 to 46 years) and an average weight of 69.48 ± 12.70 kg (ranging from 48.6 to 99.0 kg) (see Table 1).

### 3.1. Study of Bacterial Diversity

**Figure 1:**
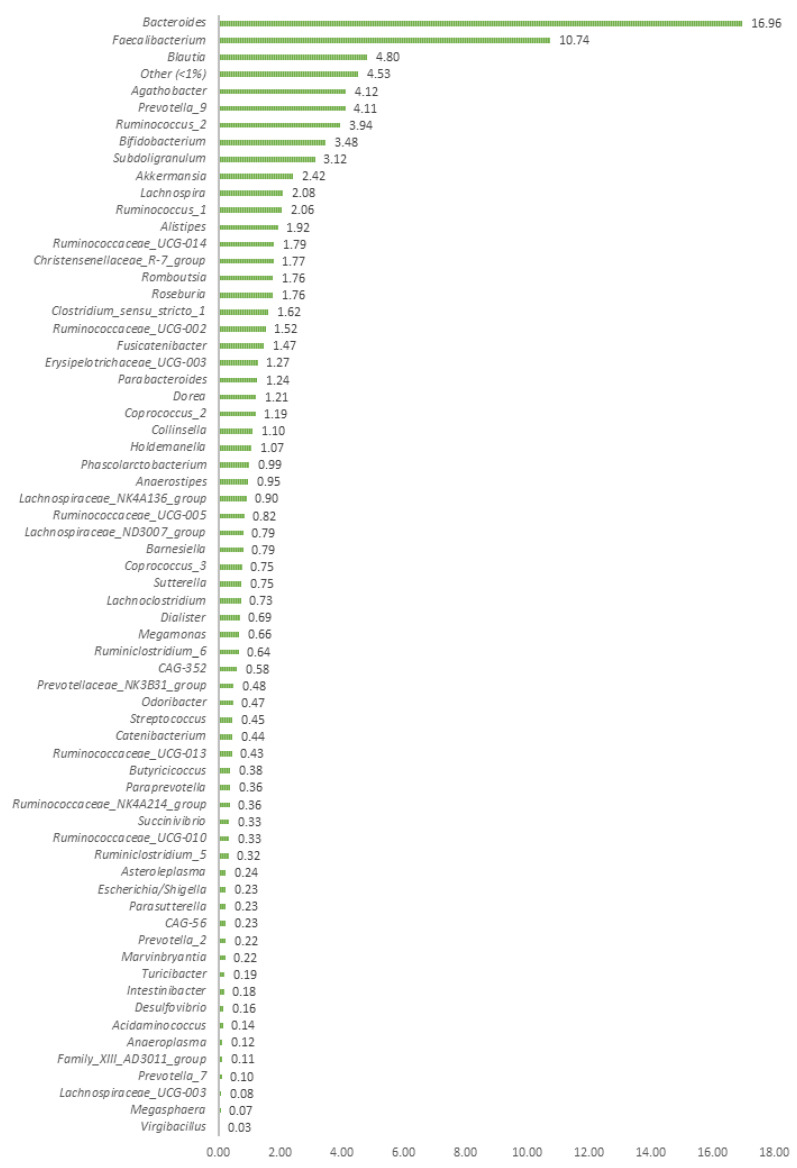
Most relevant taxa (frequency >1% in at least one sample) found in the human faecal samples of the 39 volunteers. The group “others” included the summation of the rest of the bacterial taxa with a frequency of occurrence <1.0%. Only two genera (*Bacterioides* and *Faecalibacterium*) exhibited frequencies above 10%, while the remaining taxa were widely distributed.

After filtering and performing quality depuration of the raw data obtained from the Illumina sequencing process, a total of 1,417,293 sequences were obtained from the 39 human faecal samples analysed. The average number of sequences per sample was 36,338, ranging from 29,892 to a maximum of 45,133. The bioinformatic analysis revealed the presence of a total of 235 different ASVs bacterial genera in the faecal samples (see Table S1, Supplementary Material). Before conducting the statistical analysis, bacterial ASVs present in only one subject or very low read counts in two volunteers were removed, resulting in a total of 121 ASVs for CoDA analysis (see Table S2, Supplementary Material). Among these, only 65 ASV bacterial genera at the genus level had a frequency of occurrence greater than 1% in at least one of the samples. Figure 1 depicts the average frequency of appearance of these bacterial taxa for the specific group of volunteers analysed in this clinical trial.

The alpha diversity indices were calculated using the Past program [18] according to sex (the only qualitative variable) (see Table S3, Supplementary Material). Generally, the minimum and maximum values of the indices, indicating a slightly wider range, were observed in males. However, the average values were relatively close. The number of individuals ranged from 24,253 to 45,188, with a slightly wider range for males. Dominance D was relatively low, indicating an almost similar presence of bacteria across individuals. The high values of the Simpson 1-D index also showed this trend. The Shannon H values ranged from 2.116 to 3.730, which agrees with a moderate presence of taxa and individuals. Further interpretation of the other indices can be deduced directly from Table S3 (Supplementary Material). No significant differences were observed between female and male volunteers in terms of any of the indices when comparing their values using the Kruskal–Wallis test. Thus, it can be concluded that the faeces bacterial alpha diversity among volunteers was similar regardless of sex. Additionally, the relationships between age, sex, and the alpha diversity indices were examined using Pearson’s correlation coefficient. However, no significant association was found between the alpha diversity indices and age or sex (Table S4, Supplementary Material).

### 3.2. Association of Sex, Age, and Weight with Bacteria in Faeces

As mentioned earlier, the CODA methodology in the ALDEx2 program was used for differential abundance analysis, comparing various conditions (sex, age, and weight) and the relationship with the bacterial composition obtained from faeces. The use of *glm* models allows for the analysis of complex designs. When the glm.test was applied, including all three variables (sex, age, and weight) simultaneously into the model, none was found to be statistically significant under the pval.holm (the *p*-value corrected for multiple comparisons according to the Holm–Bomberroni procedure). However, there were some interesting trends.

In particular, the variable sex exhibited significant coefficients (*p*-value < 0.05) for taxa *Senegalimassilla* (slightly higher presence in females), *Desulfovibrio* (more presence in males), and *Negativibacillus* (more abundant in females) (Table S5, Supplementary Material). The variable age showed significant coefficients for *Streptococcus*, *Tyzzerella*, *Ruminococaceae_UCG-010*, *Family_XII_UCG-001*, *Christensenellaceae_R-7_groups*, and *Ruminococaceae_UCG-005* (Table S5, Supplementary Material). Lastly, only one bacterial taxon, *Senegalimassilia*, exhibited a significant association with weight (Table S5, Supplementary Material). Although the ALDEx2 test did not identify significant effects of the studied variables on the faeces bacterial volunteers when considering the effect of multiple comparisons, the presence of a reduced number of significant variables at the usual *p*-values suggests a potential degree of association.

### 3.3. Bacterial Taxa Associated with Predictions

This analysis utilised the coda4microbiome package, which is a valuable tool for identifying the components of balances (log-ratios of taxa) through logistic or *glm* models associated with the sex, age or weight of the volunteers. For this purpose, exploratory and cross-sectional studies were performed.

**Figure 2:**
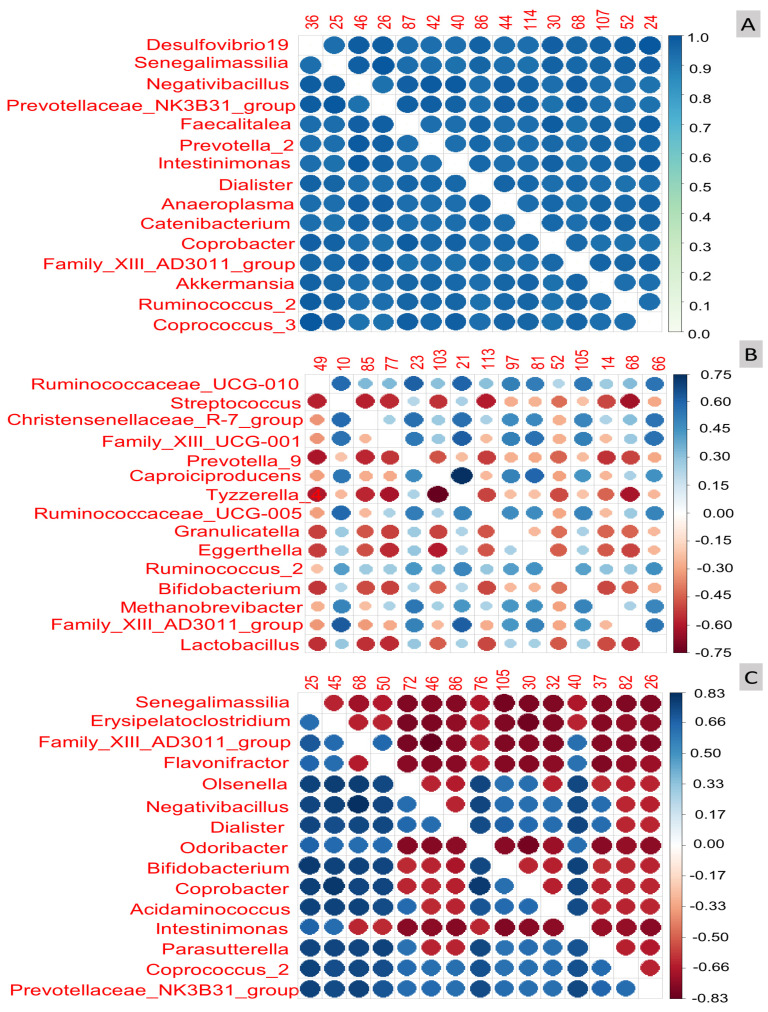
Graphical results of the exploratory analysis obtained with coda4microbiome R package for studying the human gut microbiome relationships with (**A**) Sex (prediction accuracy), (**B**) Age (Spearman correlation), and (**C**) Weight (Spearman correlation). Notice the prevalent role played by *Desulfovibrio 19*, *Senegalimassilia*, *Negativibacillus*, and the remaining genera below them for sex prediction (**A**) and the association of *Ruminococcaceae_UCG-0.10* (positively) and *Lactobacillus* (negatively, decreasing presence) with age (**B**), and the linkage of *Senegalimassilia* (negatively, declining presence) to weight (**C**).

The exploratory analysis revealed that in the cases examined, the variable sex (Figure 2A) and the balance formed by the log-ratio of the variable *Desulfovibrio* with any of the other ASVs displayed in the left column (*Senegalimassilia*, *Negativibacillus*, …, *Coprococus_3*) were able to predict the sex of the volunteers with the highest accuracy (possibly, with a *p*-value close to 1).

The remaining taxa in the left column also demonstrated strong predictive power, although slightly decreasing in overall order of importance, as indicated by their intense blue colour (located at the top of the colour scale on the right of the plot). It is important to note that not only do log-ratios of *Desulfovibrio* with any of the bacterial ASVs shown in Figure 2A predict sex with the highest accuracy, but also log-ratios formed from those ASVs below *Desulfovibrio* exhibit comparable properties due to their similar colour. The overall order of importance was as follows: *Desulfovibrio*; *Senegalimassilia*; *Negativibacillus*; *Prevotellaceae*: *NK3B31_group*; *Faecalitalea*; *Prevotella*, and so on, with the log-ratio *Prevotellaceae: NK3B31_group/Coprococcus* displaying the strongest association with sex differentiation. Hence, it can be inferred that sex likely influences the faeces bacteria, or conversely, certain balances derived from the bacterial population can accurately characterise or assign the sex of the volunteers correctly (Figure 2A).

Regarding age (Figure 2B), the strength of log-ratio linkage with the bacterial population, measured using Spearman correlation due to the *glm* model used to predict age, was not robust. The relationship rarely reached a Spearman correlation (the adequate parameter for numeric variables association) above 0.75. Notably, some of the most relevant correlations were observed between the log-ratios of *Ruminococaceae_UCG-010* vs. *Streptococcus*, *Prevotella _9K*, etc., with correlation values around 0.75 for some instances. However, compared to sex, the association with age appears to be less intense. The most important variables were *Ruminococcaceae_UCG-010*, *Streptococcus*, *Christensenellaceae_R-7_group*, *Familly: XIII_UCG-001*, and so on (Figure 2B). Additionally, the log-ratio *Tizzerella_4/Caproicidiproducents* remained strongly associated with age, as seen previously. The following order in the Pearson correlation was for the log-ratio of Streptococcus vs. most of the other taxa below it. However, the overall relationships were notably weaker, as demonstrated by numerous cells coloured with lighter tones (Figure 2B). Notice that, for numeric variables, the correlation can go from 1 (intense blue) when age and taxa follow similar trends to −1 (red) when the age and ASV follow opposed directions (negative correlation).

With respect to weight (Figure 2C), the relationship (the Spearman correlation) between this variable and the log-ratio of taxa was slightly stronger than for age, although clearly lower than for sex, and also distributed between positive and negative signs. As one descent, the list on the left of Figure 2C, the degree of association decreases, and any log-ratio involving the presence of *Senegalimassilia* is highly associated with weight. The most significant variables were *Senegalimassilia*, *Erysipelatocclostridium*, *Family_XIII_AD3011_group*, and so on (Figure 2C), with the log-ratio *Family_XIII_AD3011_group/Negativibacillus* showing the highest association with weight.

Thus, the exploratory analysis has revealed a strong relationship between specific log-ratios and the variables, indicating that the association between the human faecal bacteria could be broader than what is typically identified by ALDEx2, which primarily focuses on disclosing significant differential abundances typically above relevant thresholds. The differences in the gut microbiome due to the variables under study could be subtler.

**Figure 3:**
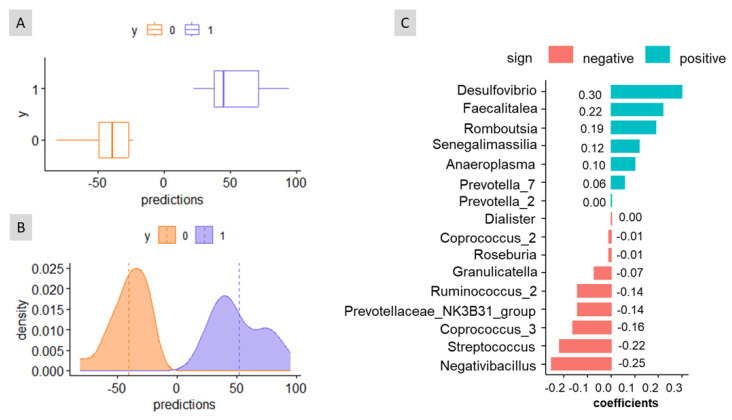
Graphical results of the cross-sectional study obtained with coda4microbiome R package for the balance of taxa as a function of variable sex. (**A**) Boxplot of predictions. (**B**) Distribution of predictions. (**C**) Balance signature for sex prediction. As deduced from (**C**), the increased presence of ASVs in the numerator leads to higher balance values associated with males (on the right in (**A**,**B**)); on the contrary, the abundant presence of those ASVs in the denominator would characterise the female sex (on the left in (**A**,**B**)).

In a cross-sectional study, when sex was assigned without considering the effects of age and weight, the analysis resulted in non-overlapping distributions of the prediction curves (Figure 3A,B). The algorithm also provides three classification accuracy measures: the apparent AUC (the AUC of the signature applied to the same data that were used to generate the model, 1.00 in this case), and the mean and the standard deviation of the cross-validation AUC (1.37 and 0.14, respectively). This suggests the possibility of assigning sex without controlling for age or weight. The balance signature associated with this sex prediction included a small number of bacterial taxa (Figure 3C). In the numerator, the balance consisted of *Desulfovibrio*, *Faecalitalea*, *Romboutsia*, *Senegalimassilia*, *Anaeroplasma*, *Prevotella_7*, and *Prevotella_2* (coeff < +0.005) while in the denominator, it included *Dialister* (coeff. < −0.005), *Coprococcus_2*, *Roseburia*, *Granulicatella*, *Ruminococcus_2*, *Prevotellaceae_NK3B31_group*, *Coprococcus_3*, *Streptococcus*, and *Negatibacillus*. Based on Figure 3C, the bacterial taxa in the denominator would, overall, predominate in females, as indicated by the left boxplot and distribution curves. The analysis also considered age and weight as co-variates but yielded similar results. Thus, this study demonstrated that age and weight have limited relevance for assigning sex based on the faecal bacteria composition, at least within the group of volunteers analysed in this study.

**Figure 4:**
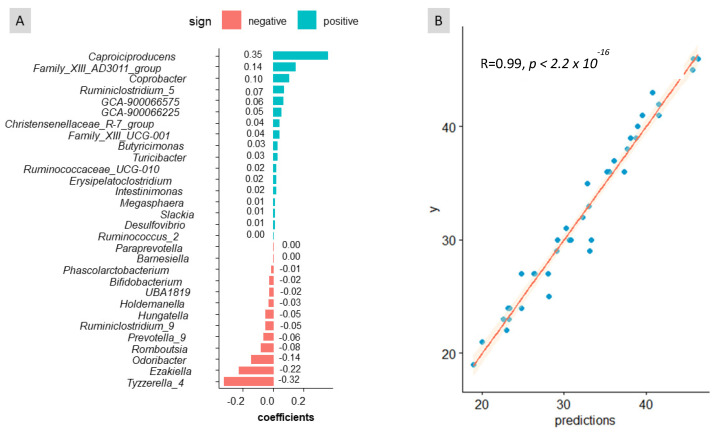
Graphical results of the cross-sectional study obtained with coda4microbiome R package for the balance of taxa as a function of variable age. (**A**) Balance signature for sex prediction. (**B**) Experimental vs. age predictions. One can deduct from (**A**) that the increased presence of ASVs in the numerator leads to higher balance values associated with older people; on the contrary, the abundance of those ASVs in the denominator will lead to lower balances linked to younger people.

Considering the important role played by sex in the gut microbiota, predictions of age and weight were performed using sex as a co-variable. The balance signature inferred for age (Figure 4A) included numerous bacterial taxa, most of which had low relevance. Noteworthy contributors to this balance were *Caproiciproducens*, *Family_XIII_AD3011_group* in the numerator and *Odoribacter*, *Ezakiella*, and *Tyzzerella_4* in the denominator. Overall, this balance signature differed considerably from the one selected for sex. This analysis showed a good agreement between the observed and predicted values (Figure 4B), with an R-square of 0.989. This study’s findings reveal that age influences the composition of faecal microbiota. Although estimating age solely from the faeces microbiome is unlikely, it was interesting to demonstrate its potential when accounting for the effects of sex and weight. Furthermore, the results obtained when using sex and weight as co-variables were similar to those obtained when considering sex alone. This provides additional evidence for the limited influence of weight (within limits used in the experiment) on the composition of the faecal bacteria.

Due to the apparent relatively low effect of weight on the faecal bacterial composition among the volunteers, its prediction was only possible when age was used as a co-variable. However, even in this scenario, the results were unreliable (see Figure S1, Supplementary Material).

## 4. Discussion

Previous research has indicated that various factors, such as diet, exercise, sex, age, or weight, can influence the diversity and richness of gut bacteria in humans [22,23,24,25,26,27]. Specifically, Cuesta-Zuluaga et al. [28] investigated the association of age, sex, and gut bacterial alpha diversity in three large cohorts of adults. They further attempted to predict the microbiota composition in individuals using a machine-learning approach. Following this line, this work aims to examine the faecal bacterial composition of this specific group of the Mediterranean population in relation to sex, age, and weight, utilising CoDA methodology.

It has been reported that many of the over 2000 human-associated microbial species identified can be classified into 12 different phyla, with approximately 93% belonging to *Actinobacteria*, *Bacteroidetes*, *Proteobacteria*, and *Firmicutes* phylum [29,30]. Specifically, the human gut microbiome exhibits lower taxonomically diversity than other microbial communities, such as the skin, and has a high level of functional redundancy [31,32]. In this work, the 235 bacterial ASVs detected were primarily classified into the phyla *Firmicutes*, *Actinobacteria*, *Proteobacteria*, and *Bacteroidetes*, albeit there were also diverse genera assigned to less abundant phyla, such as *Synergistetes*, *Fusobacteria*, *Euryarchaeota*, *Epsilonbacteraeota*, and *Verrucomicrobia* (see Table S1, Supplementary Material). Among the detected genera, *Bacteroides* was the most frequently detected genus (average 16.96%), followed by *Faecalibacterium* (10.74%), *Blautia* (4.80%), *Agathobacter* (4.12%), *Prevotella_9* (4.11%), and *Ruminococcus_2* (3.94%) (Figure 1). The presence of the three most abundant genera (*Bacteroides*, *Faecalibacterium*, and *Blautia*) in the faeces of this specific group of volunteers is generally considered beneficial [33,34].

Results obtained in this work partially agree with the findings of Cuesta-Zuluaga et al. [28], who recently investigated the association of age, sex, and gut bacterial alpha diversity in many people from the United States and the United Kingdom. In three of the four cohorts analysed, they found a robust positive association between age and alpha diversity in young adults, which reached a plateau after the age of 40 years. They also reported sex-dependent differences, more pronounced in younger than middle-aged adults, with women having higher alpha diversity than men. However, in our study, analysing the 39 Andalusian individuals included in the clinical trial, no significant associations were found between alpha diversity indices and age or sex, consistent with the results observed by Cuesta-Zulaga et al. [28] for the four cohorts analysed (Chinese population).

According to the results obtained through CoDA methodology, the log-ratios of specific bacterial genera exhibited a strong association with the sex of the volunteers. In contrast, the association with age and weight was less pronounced. Previous studies have demonstrated that gut microbiota diversity was influenced by various factors, including sex differences that become more apparent after puberty [35,36]. Hormone levels have been associated with specific compositions of gut bacterial microbiota, as evidenced by various studies conducted on healthy women and men [24,25,37]. A recent review by Yoon and Kim [26] investigated the relationship between specific sex hormones and a higher abundance of *Desulfovibrio* in rats and mice. Although *Desulfovibrio* was detected as a minor genus in this study, with an average appearance frequency of 0.16%, CoDA revealed that this genus played a relevant role in predicting the sex of the volunteers for this specific group of people.

We now have a better understanding of the profound impact of the gut microbiome on energy balance, with diverse mechanisms influencing both aspects. This growing knowledge of microbial contributions to energy metabolism presents new opportunities for weight management [27]. In a recent study, Maslennikov et al. [38] reported a decrease in the abundance of *Coprococcus*, *Desulfovibrio*, and *Senegalimassilia* in the gut microbiome of patients with body cell mass deficiency. *Desulfovibrio* has also been negatively correlated with body mass index, waist size, triglyceride and uric acid levels, indicating a potential association with host health [39].

Regarding other aspects of human health and specific faecal bacterial genera detected in this study, *Senegalimassilia* has been identified as a protective factor of the gut microbiome against hypertension [40]. The abundance of *Negativibacillus* was found to decrease in lactose-intolerant individuals, suggesting its potential involvement in lactose utilisation [41]. *Tyzzerella* and *Coprococcus* were enriched in the faeces of people with a high risk of cardiovascular disease, while *Ruminococcus* was present in higher proportions in people at low risk [42].

## 5. Conclusions

This study successfully characterised the typical faecal bacterial composition in 39 volunteers from the Córdoba and Sevilla provinces (Andalusia, Spain). The analysis revealed a high level of alpha diversity without significant influence from the sex, age, or weight of the participants. However, employing CoDA, certain bacterial taxa exhibited marked trends for differential abundance, primarily driven by sex, age, and weight. The balance signatures involved only a reduced number of bacterial taxa, which demonstrated the potential for accurate sample assignment to the corresponding sex or predicting the age of the volunteers with moderate error. Therefore, the application of CoDA serves as a practical methodology for studying the influence of variables on the composition of the human gut microbiome, albeit further studies should be carried out with a greater number of participants to validate these results.

## Figures and Tables

**Table 1:** Descriptive statistics for sex, age (years) and weight (kg) obtained from the 39 volunteers included in the clinical trial. Table S2 (supplementary material) shows the age and weight of each specific individual included in this study.

Variable	Type	Mean	Confidence −95%	Confidence +95%	Standard Deviation	Median	Minimum	Maximum
Sex	Qualitative *	-	-	-	-	-	-	-
Age	Quantitative	32.51	30.03	34.99	7.64	31.00	19.00	46.00
Weight	Quantitative	69.48	65.35	73.62	12.74	66.40	48.60	99.00

* Binary variable, with a total of 20 females and 19 males.

## Data Availability

The data and biological samples supporting this study’s findings are available from the corresponding author upon reasonable request to favour new collaborations.

## Supplementary Materials

The following supporting information can be downloaded at: https://www.mdpi.com/article/10.3390/biomedicines11082134/s1, Figure S1: CoDA for human faecal bacteria study and their relationships with sex, age, and weight. Predictions obtained by coda4microbiome for weight, using age as covariable; Table S1: CoDA for human faecal bacteria study and their relationships with sex, age, and weight. Frequency of all ASV bacterial genera detected by the metataxonomic analysis in the 39 volunteers included in this study; Table S2: CoDA for human faecal bacteria study and their relationships with sex, age, and weight. The reduced data set was used for CoDa analysis. It shows the number of sequences according to volunteers and taxa. For the analysis, the presence of zeros was substituted by values imputed according to the program requirements (Bayesian multiplicative methods or replaced by 1; Table S3: CoDA for human faecal bacteria study and their relationships with sex, age, and weight. Values of the alpha-diversity indices according to sex; Table S4. CoDA for human faecal bacteria study and their relationships with sex, age, and weight. Relationships between age and weight of individuals with the alpha-biodiversity indices as studied via Pearson’s correlation. Furthermore, it also includes the relationships among the awn alpha-biodiversity indices; Table S5: CoDA for human faecal bacteria study and their relationships with sex, age, and weight. Significant bacterial taxa (marked yellow) selected by the ALDEx2 program using the glm.test for variables sex (A), age (B), and weight (C).
